# Prediction of haplotypes for ungenotyped animals and its effect on marker-assisted breeding value estimation

**DOI:** 10.1186/1297-9686-42-10

**Published:** 2010-03-22

**Authors:** Han A Mulder, Mario PL Calus, Roel F Veerkamp

**Affiliations:** 1 Animal Breeding and Genomics Centre, Wageningen UR Livestock Research, PO Box 65, 8200 AB Lelystad, The Netherlands

## Abstract

**Background:**

In livestock populations, missing genotypes on a large proportion of animals are a major problem to implement the estimation of marker-assisted breeding values using haplotypes. The objective of this article is to develop a method to predict haplotypes of animals that are not genotyped using mixed model equations and to investigate the effect of using these predicted haplotypes on the accuracy of marker-assisted breeding value estimation.

**Methods:**

For genotyped animals, haplotypes were determined and for each animal the number of haplotype copies (nhc) was counted, i.e. 0, 1 or 2 copies. In a mixed model framework, nhc for each haplotype were predicted for ungenotyped animals as well as for genotyped animals using the additive genetic relationship matrix. The heritability of nhc was assumed to be 0.99, allowing for minor genotyping and haplotyping errors. The predicted nhc were subsequently used in marker-assisted breeding value estimation by applying random regression on these covariables. To evaluate the method, a population was simulated with one additive QTL and an additive polygenic genetic effect. The QTL was located in the middle of a haplotype based on SNP-markers.

**Results:**

The accuracy of predicted haplotype copies for ungenotyped animals ranged between 0.59 and 0.64 depending on haplotype length. Because powerful BLUP-software was used, the method was computationally very efficient. The accuracy of total EBV increased for genotyped animals when marker-assisted breeding value estimation was compared with conventional breeding value estimation, but for ungenotyped animals the increase was marginal unless the heritability was smaller than 0.1. Haplotypes based on four markers yielded the highest accuracies and when only the nearest left marker was used, it yielded the lowest accuracy. The accuracy increased with increasing marker density. Accuracy of the total EBV approached that of gene-assisted BLUP when 4-marker haplotypes were used with a distance of 0.1 cM between the markers.

**Conclusions:**

The proposed method is computationally very efficient and suitable for marker-assisted breeding value estimation in large livestock populations including effects of a number of known QTL. Marker-assisted breeding value estimation using predicted haplotypes increases accuracy especially for traits with low heritability.

## Background

In livestock, many QTL regions have been identified for quantitative traits [1]. In some cases, fine mapping has also led to the detection of causative mutations, e.g. DGAT1 in dairy cattle for milk yield and milk composition [2,3] and IGF2 in pigs for body weight [4]. In breeding programs these QTL-regions can be utilized in marker-assisted selection (MAS). Three types of markers can be used: markers in linkage equilibrium with the QTL (LE-MAS), markers in linkage disequilibrium with the QTL (LD-MAS) and the causative mutation itself as in gene-assisted selection (GAS). GAS leads to the highest genetic gain, because no recombination exists between the marker and QTL [5]. However, identifying the gene is not easy and is resource demanding [1]. The amount of QTL variation explained by markers in LD-MAS can be increased by increasing the marker density and thereby increasing the LD between markers and QTL. Alternatively, combining alleles of different marker loci into haplotypes is expected to increase the proportion of captured QTL variance as well. Based on data of a whole genome scan with 9323 SNP-markers in Angus cattle, Hayes *et al*. [6] have reported that 4 and 6-marker haplotypes increased the accuracy of MAS more than the single marker in highest LD with the QTL. However, 2-marker haplotypes performed worse than the best marker.

One of the challenges when applying MAS in livestock populations is that often a large part of the population is not genotyped, i.e. some animals have only phenotypes, some have only genotypes and others have both genotypes and phenotypes. Several methods have been proposed to overcome these differences. For LE-MAS, one would like to apply a method that uses identity-by-descent (IBD) information of haplotypes to properly account for relationships between haplotypes of related animals and to account for phase differences between markers and QTL in different families [7]. Creation of inverse IBD-matrices is, however, very time consuming [8]. With high-density SNP-chips, LD-MAS can be applied without having to use IBD-matrices. With LD-MAS, either flanking markers or identical-by-state haplotypes (IBS) can be used in marker-assisted breeding value estimation. When using flanking markers in MAS, genotype probabilities could be calculated with iterative peeling methods [9-13] but these are time consuming. Gengler *et al*. [14,15] have proposed a straightforward and quick method to predict genotype probabilities and gene contents for bi-allelic markers using a mixed model methodology, where gene content is the number of positive (negative) alleles (i.e. 2, 1, 0 for AA, Aa, aa). For ungenotyped animals, the accuracy of predicted gene contents is similar whether mixed model equations or single-marker iterative peeling are used [8,14]. Gengler *et al*. [14] suggested that the method can also be applied in the case of multi-allelic markers. Multi-marker IBS haplotypes can be considered as a special form of multi-allelic markers, making the mixed model methodology a candidate method to predict haplotypes for ungenotyped animals.

The objective of this article is to develop a method to predict haplotypes of animals that are not genotyped using mixed model equations and to investigate the effect of using those predicted haplotypes on the accuracy of marker-assisted breeding value estimation. The method is evaluated using Monte Carlo simulation, varying haplotype length, heritability of the trait and distance between the markers. The method is compared to gene-assisted and conventional breeding value estimation, which yield, respectively, the upper and lower limit of accuracy.

## Methods

### Prediction of haplotypes with missing genotypes

Consider a situation where a QTL-region is mapped for a trait, without having identified the causative mutation and where some animals in the population are genotyped for SNP-markers in that region, but most of them are not genotyped, which is very common in animal breeding populations. In this study we would like to use IBS-haplotypes in marker-assisted breeding value estimation. When the haplotype is based on the single SNP-marker closest to the QTL, the method of Gengler *et al*. [14,15] can be used to predict the missing 'gene content', the number of A-alleles, if there are A and a-alleles. The method of Gengler *et al*. [14,15] uses the additive genetic relationship matrix in a mixed model setting to predict the gene contents of those animals not genotyped based on genotyped relatives. This method can not be applied directly for haplotypes based on multiple markers, because discrete haplotypes can not be directly constructed based on predicted continuous gene contents of SNP-markers for ungenotyped animals. However, this procedure can be easily modified to apply to a situation with haplotypes based on multiple markers. Consider that haplotypes are based on two bi-allelic markers, one on each side of the QTL. There are four possible haplotypes. For every genotyped animal, one can infer how many copies it carries for each haplotype (*nhc *= number of haplotype copies), which is 0, 1 or 2 (see Table 1 for a small example). This is in essence the same as the 'gene content' for a bi-allelic locus and the same mixed model methodology with the additive genetic relationship matrix can be applied to predict the *nhc *for each haplotype for the ungenotyped animals. In the case of *n *haplotypes this can be modeled as:(1)

**Table 1:** Example with four animals with the number of haplotype copies for two SNP-marker haplotypes

			Number of haplotype copies (*nhc*)			
			
Animal	Haplotype 1	Haplotype 2	Hap1 (11)	Hap2 (12)	Hap3 (21)	Hap4 (22)
1	11	11	2	0	0	0
2	11	12	1	1	0	0
3	11	21	1	0	1	0
4	11	22	1	0	0	1

where *nhc*_*i *_is the number of copies of haplotype *i *(which is 0, 1 or 2 effectively),  is the population mean number of copies of haplotype *i*, *d*_*i *_is the EBV for *nhc*_*i *_and  is the residual of *nhc*_*i*_. Although  for each animal, it is assumed that the haplotypes are independent from each other; therefore *n *univariate mixed model analyses can be performed. Analogous to gene contents for a bi-allelic locus [14], this can be formulated in mixed model matrix notation as:(2)

where **1 **is a vector of ones, **M **is a design matrix linking **d **with **nhc**_*y*_, **A**^**-1 **^is the inverse additive genetic relationship matrix, *λ *is the variance ratio of residual variance and additive genetic variance for *nhc *allowing for a small proportion of genotyping and haplotyping errors or recombination , **d **is a vector with the EBV for *nhc *with **d**_*y *_for genotyped animals and **d**_*x *_for ungenotyped animals, **nhc**_*y *_is a vector with observed *nhc *of genotyped animals and is set to missing for ungenotyped animals. The heritability assumed for *nhc *is 0.99. Basically, with no genotyping or haplotyping errors,  (the predicted *nhc*) should be equal to the phenotype (the true *nhc*) for genotyped animals, implying a heritability of 1.0. In the case of haplotypes, recombinant haplotypes can be transmitted from one parent to its offspring. In such a case, the recombinant haplotype can not be fully explained in the model by the haplotypes of the parent. This decreases the parent-offspring regression, i.e. decreasing the heritability. Here we set the heritability to 0.99 to allow for some small proportions of genotyping and haplotyping errors and recombination. Preliminary analysis showed no effect when the heritability was changed to 0.95.

### Marker-assisted breeding value estimation using predicted haplotypes

To include the effects of the haplotypes to perform marker-assisted breeding value estimation using best linear unbiased prediction (MABLUP), these *nhc *can be used as covariables in random regression, where inclusion as a random effect is preferred so that effects will be regressed towards zero when there is hardly any phenotypic information, e.g. a certain haplotype appears only in one animal with a phenotypic record. Assuming no other systematic environmental effects, the model is as follows:(3)

where *y *is the phenotype, *μ *is the overall mean and modeled as a fixed effect, *u*_*pol *_is the random polygenic EBV, , which is the predicted number of copies of haplotype *i*, *h*_*i *_is the random regression coefficient for haplotype *i *and *e *is the residual. In matrix notation the model can be summarized as:(4)

where **X **and **Z **are the design matrices for fixed effects and polygenic breeding values, respectively, the matrix **W **contains the  for all haplotypes, *λ*_*pol *_and *λ*_*h *_are respectively the variance ratios for the polygenic breeding values and the random regression on , **b **is the vector with solutions for fixed effects (in this case only the mean), **u**_*pol *_is the vector with *u*_*pol *_and **h**_*i *_is the vector with *h*_*i*_. The variance of *h*_*i *_is  (see Appendix for derivation), where  is the additive genetic QTL-variance, and the variance of *u*_*pol *_is , where  is the additive genetic variance due to the polygenic effect. Equations (3) and (4) can be considered as a generalization of the method by Gengler *et al*. [14,15] to multi-allelic markers and haplotypes.

### Evaluation of method

#### Simulation

Monte Carlo simulation was used to evaluate the method. The simulation scheme represented a nested full-sib half-sib design (multiple offspring per mating and dam nested within sire) with discrete generations which is common in commercial animal breeding programs. The simulation scheme was identical to that reported in Mulder *et al*. [8]. One trait was simulated with additive genetic effects of one bi-allelic QTL *A*_*qtl*_, a polygenic additive genetic effect *A*_*pol *_and a residual effect *e *(*P *= *A*_*qtl *_+ *A*_*pol *_+*e*). All animals had phenotypic records. Because the method of MABLUP relies on linkage disequilibrium (LD) between markers and QTL, first, 100 generations of random mating were performed prior to the data collection scheme (generation 101 - 105).

In the first 100 generations, 50 sires and 50 dams were randomly mated each generation. The QTL and 20 bi-allelic markers were placed on one 1 M long chromosome. The QTL was placed in the middle of the chromosome and the markers were equally spaced, their distance varying from 0.1 to 5 cM. The QTL was in the middle of the marker bracket between marker 10 and 11. In the founder generation, all markers and the QTL were in linkage equilibrium and had a fixed allele frequency of 0.5. The QTL-variance  was set to 15% of the total genetic variance, when the allele frequency is 0.5. The allele substitution effect was set to , assuming that the allele frequencies *p *and *q *are 0.5, which is the case in the founder generation. Recombination rates were calculated using Haldane's mapping function [16]. During these 100 generations, some markers or the QTL became fixed due to drift.

After establishing LD, from generation 101 onwards and for each generation 50 sires and 250 dams were selected based on conventional BLUP-EBV (Equation (3) without haplotype effects) and randomly mated to produce 2,000 offspring. Each sire was mated to five dams and each dam produced four male and four female offspring, resulting in that each sire had 40 half-sib offspring, five full-sib groups of eight full-sibs. A total of five generations of phenotypic data (generation 101 - 105) were created and used in breeding value estimation (10,000 animals in total). The animals of generation 101 served as base generation in the pedigree. The generations 102 - 104 were used to create linkage disequilibrium due to selection [17].

In generation 101, simulated polygenic effects were sampled from *N*(0, ), where  is the polygenic genetic variance. In subsequent generations polygenic effects were sampled from *N*(0.5 *A*_*pol*, *s *_+ 0.5 *A*_*pol*, *d*_, 0.5  (1 - *f*_*p*_)), where *f*_*p *_is the average inbreeding coefficient of the parents. Inbreeding coefficients were calculated using the Meuwissen and Luo [18] algorithm. Residual effects were sampled from *N*(0, ), where  is the residual variance.

The overall heritability was set to 0.03, 0.10 or 0.30, while the QTL explained 15% of the total genetic variance when the allele frequency was 0.5 as it was in the founder generation. The phenotypic variance was 1.0 in all situations when the allele frequency of the QTL was 0.5. The realized variance of the QTL was lower due to deviations of the allele frequency from 0.5 and re-estimated in generation 101. Results were based on 200 effective replicates after discarding the replicates with minor allele frequency of the QTL in the last generation (generation 105) less than 0.05. Averaged over all effective replicates, the average allele frequency of the negative QTL-allele was 0.63 in generation 101 before selection started and deviated from 0.5, because in replicates with allele frequencies closer to 0, the QTL was more likely to become fixed in generations 101-105 due to selection. The used parameter values are listed in Table 2.

**Table 2:** Parameter values for simulation

Parameter	Default value	Alternative values
Number of sires per generation	50	
Number of dams per generation	250	
Total number of animals	10,000	
Number of progeny per dam	8	
Number of generations	5	
Heritability	0.3	0.03 and 0.10
Proportion of genetic variance explained by QTL	0.15	
Number of markers simulated	20	
Distance between markers	0.1 cM	0.5, 1.0, 2.0, 5.0 cM
Number of markers used	10	
Number of replicates	200	

#### Haplotype methods used for marker-assisted breeding value estimation

In this study we used three types of haplotypes: 1) the closest neighboring left marker of the QTL is used as a single-marker haplotype (NM), 2) both flanking markers closest to the QTL-locus are used to form a 2-marker haplotype (HAP2) and 3) on both sides the two markers closest to the QTL are used to form a 4-marker haplotype (HAP4). In the case of NM, Equation (3) and (4) reduced to the method by Gengler *et al*. [14,15] with the difference that in this case it was not the causative mutation, but a linked marker. In addition,  = *α*^2^, where *α *is the allele substitution effect (see equation A1 in the Appendix), because we modeled only one SNP marker allele. The markers chosen to form haplotypes had minor allele frequencies of at least 5% in generation 105. Haplotypes were known from the simulation and thus, phasing was not needed.

#### Genotyping and breeding value estimation

In generation 105, the breeding program starts with MABLUP according to Equation (3) and (4) using the three different haplotype methods. We simulated three genotyping scenarios: (1) only sires and males in the last generation are genotyped and (default) (2) all males are genotyped and (3) all animals are genotyped. In scenario 1 and 2, females are not genotyped. In addition to MABLUP, gene-assisted BLUP (GABLUP) and conventional BLUP (CONBLUP) are also performed for comparison. For GABLUP, it is assumed that all animals are genotyped for the QTL. For GABLUP the model is equal to Equation (3), with the difference that the true gene content is used as *nhc *and the variance is the same as for NM. For CONBLUP, Equation (3) is used without regression on *nhc *and the variance of the additive genetic effect is set to . For all evaluations, mixed model equations were solved using MiX99, which makes use of the preconditioned conjugate gradient algorithm [19]. The mixed model equations were considered converged when the relative difference between the left-hand and right-hand sides of the mixed model equations was smaller than 1.0 * 10^-10^.

Accuracies were calculated as correlations between estimated and true breeding values. The QTL-EBV was calculated as  for each animal. The total EBV was calculated as the sum of the QTL-EBV and the polygenic EBV. Accuracies of MABLUP were compared to those of GABLUP and CONBLUP. The accuracies of GABLUP and CONBLUP can be considered as the upper and lower limits for the MABLUP accuracy. In addition, regressions of true breeding values on estimated breeding values were calculated to get an idea of the over- (regression coefficient < 1.0) or underestimation (regression coefficient > 1.0) of the variance of EBV. Bias of estimated breeding values was calculated as estimated breeding values minus true breeding values. In addition, accuracies of  were calculated as correlations between estimated and true *nhc *and regressions of true on estimated *nhc *were calculated.

#### Proportion of QTL-variance explained by the haplotypes

The proportion of QTL-variance explained by the three different haplotypes NM, HAP2 and HAP4 was calculated to assess whether using IBS-haplotypes was suitable. The proportion of QTL-variance explained by the haplotypes is also a measure of linkage disequilibrium between the haplotype and the QTL. For NM, the *r*^2 ^between the marker and the QTL can be calculated as the squared correlation between them [20]. For multi-allelic haplotypes, such as HAP2 and HAP4, *r*^2 ^was calculated according to Equation (2) in Hayes *et al*. [6], based on an equation for multi-allelic markers by Zhao *et al*. [21].

## Results

### Analysis of haplotypes

#### Statistics of predicted number of haplotype copies

Table 3 shows the mean, standard deviation and mean square error (MSE) for predicted number of haplotype copies (*nhc*) for ungenotyped animals as a function of the true number of haplotype copies. For all three methods, the predicted *nhc *increased with the true *nhc *and a clear distinction was made in *nhc *between animals carrying the haplotype or not. For genotyped animals the predicted *nhc *closely resembled the true *nhc*. For ungenotyped animals, the absolute numbers decreased from NM towards HAP4, due to regression to the mean and the mean *nhc *decreased from NM towards HAP4, albeit the difference between homozygotic carrier and non-carrier is largest for HAP4. As a consequence, the MSE increased with increasing true *nhc *for HAP2 and HAP4 and for HAP4 more than for HAP2. In general, the mean *nhc *decreased with the frequency of the haplotype (results not shown).

**Table 3:** Summary statistics of predicted number of haplotype copies for ungenotyped animals

Haplotype method	True *nhc*	Mean	SD	MSE
NM	0	0.59	0.08	0.54
	1	0.99	0.09	0.20
	2	1.43	0.08	0.52
HAP2	0	0.34	0.06	0.27
	1	0.76	0.08	0.24
	2	1.24	0.08	0.75
HAP4	0	0.16	0.04	0.11
	1	0.58	0.06	0.32
	2	1.13	0.08	0.90

Mean, standard deviation (SD) and mean square error (MSE) of predicted number of haplotype copies (*nhc*) for neighboring marker (NM), 2-marker haplotype (HAP2) and 4-marker haplotypes (HAP4) for ungenotyped animals in the last generation (females) as a function of true *nhc *(sires and males in last generation are genotyped; distance between markers is 0.1 cM, heritability is 0.30, the QTL explains 15% of the genetic variance, results are averages of 200 replicates)

Table 4 shows the accuracy of predicted *nhc *and the regression of true *nhc *on predicted *nhc *for ungenotyped females. The accuracy decreased from NM towards HAP4, especially for HAP4, due to recombination between genotyped ancestors and ungenotyped offspring. Especially for HAP4, the accuracy decreased when the marker distance increased, which is again due to a higher probability of recombination (results not shown). The regression of true *nhc *on predicted *nhc *was approximately 1 for NM and HAP2, but somewhat lower for HAP4, due to the lower accuracy.

**Table 4:** Accuracy and regression coefficients of predicted number of haplotype copies for ungenotyped animals

Haplotype method	Accuracy *nhc *(se)		Regression^1 ^true *nhc *on predicted *nhc *(se)	
NM	0.643	(0.003)	1.005	(0.004)
HAP2	0.630	(0.007)	0.994	(0.022)
HAP4	0.595	(0.012)	0.914	(0.038)

Accuracy of number of haplotype copies (*nhc*) and regression of true *nhc *on predicted *nhc *for neighboring marker (NM), 2-marker haplotype (HAP2) and 4-marker haplotypes (HAP4) for ungenotyped animals in the last generation (females) (sires and males in last generation are genotyped; distance between markers is 0.1 cM, heritability is 0.30, the QTL explains 15% of the genetic variance, results are averages of 200 replicates)

^1^Regressions where the variance of the predicted *nhc *was smaller than 0.0001 were omitted (denominator of regression coefficient)

#### Proportion of QTL-variance explained by haplotype

**Figure 1:**
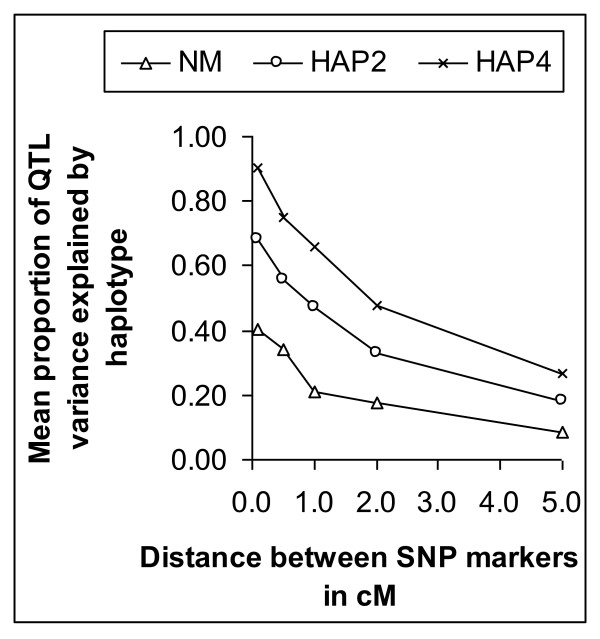
**Mean proportion of QTL-variance explained by haplotypes as a function of distance between SNP-markers**. Mean proportion of QTL-variance explained by neighboring marker (NM), 2-marker haplotype (HAP2) and 4-marker haplotype (HAP4); average of 200 replicates.

**Figure 2:**
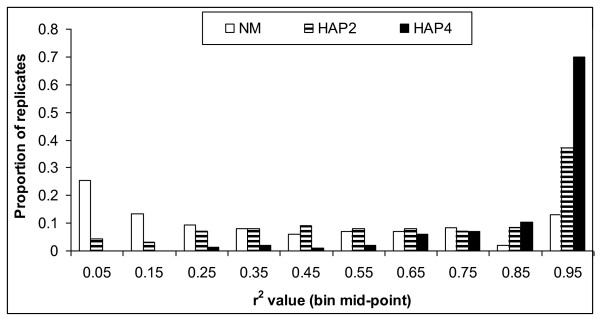
**Frequency distribution of QTL-variance explained by haplotypes**. Proportion of replicates per 0.1-bin class of proportion of QTL variance (r^2^) explained by neighboring marker (NM), 2-marker haplotype (HAP2) and 4-marker haplotype (HAP4); average of 200 replicates; sires and males in last generation are genotyped; distance between markers is 0.1 cM.

Figure 1 shows the mean proportion of QTL variance (r^2^) explained by the haplotype as a function of marker distance. For all three methods, r^2 ^decreased with increasing marker distance. The HAP4 method captured most of the QTL variance and NM the least. Figure 2 shows the frequency distribution of r^2 ^values for the three methods at a marker density of 0.1 cM. It shows that HAP4 had the highest proportion of replicates with r^2 ^values between 0.90 and 1.00. With NM and HAP2, a substantial proportion of replicates had r^2 ^values below 20% indicating that the haplotype explained very little QTL-variance.

### Accuracy of EBV

#### Effect of genotyping scenario

Table 5 shows the accuracies of QTL-EBV, polygenic EBV and total EBV for genotyped males and ungenotyped females under different genotyping scenarios with the three methods of MABLUP when the marker distance was 0.1 cM. The accuracy of polygenic and total EBV hardly changed when the number of genotyped animals increased. The accuracy of QTL-EBV increased only slightly with an increasing number of genotyped animals. This means that the use of predicted haplotypes in MABLUP did not negatively affect the accuracy of EBV. Because of the small differences in accuracy, in the rest of the article we only show results under the scenario where sires and males in the last generation were genotyped.

**Table 5:** Accuracy of EBV for genotyped males and ungenotyped females in different genotyping scenarios

		Genotyped			Ungenotyped		
			
EBV	Scenario^2^	NM	HAP2	HAP4	NM	HAP2	HAP4
QTL	sires + males last	0.534	0.775	0.912	0.336	0.491	0.580
	all males genotyped	0.534	0.774	0.926	0.337	0.493	0.591
	all genotyped	0.534	0.776	0.932			
Polygenic	only sires + males last	0.567	0.576	0.583	0.566	0.575	0.582
	all males genotyped	0.567	0.577	0.584	0.566	0.576	0.583
	all genotyped	0.567	0.578	0.586			
Total	only sires + males last	0.605	0.616	0.622	0.595	0.596	0.596
	all males genotyped	0.605	0.616	0.624	0.595	0.596	0.596
	all genotyped	0.606	0.617	0.625			

Accuracies^1 ^of QTL-EBV, polygenic EBV and total EBV for different genotyping scenarios for marker-assisted BLUP with neighboring marker (NM), 2-marker haplotype (HAP2) and 4-marker haplotypes (HAP4) (distance between markers is 0.1 cM, heritability is 0.30, the QTL explains 15% of the genetic variance, results are averages of 200 replicates)

^1^Standard errors were between 0.005 and 0.021 for QTL_EBV, between 0.002 and 0.003 for polygenic and total EBV; ^2 ^in the first scenario sires from generation 101-104 and males in generation 105 were genotyped (1,200 genotyped animals); in scenario 2 all males were genotyped (5,000 genotyped animals) and in the last scenario all animals are genotyped (10,000 genotypes)

#### Effect of marker density

**Figure 3:**
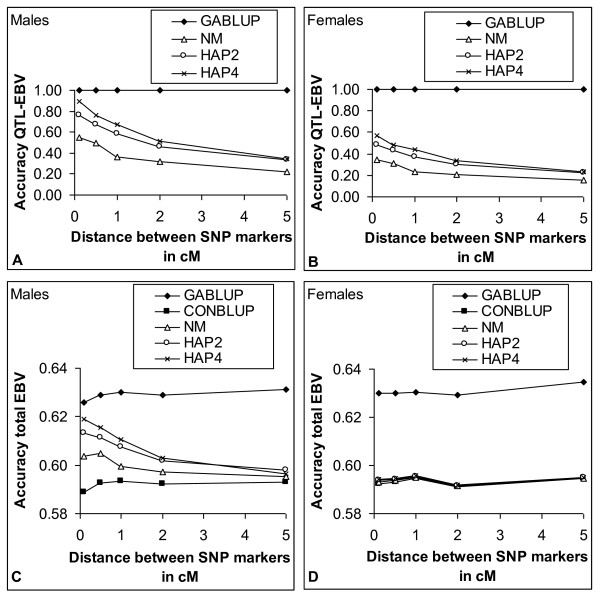
**Accuracy of QTL-EBV and total EBV as a function of marker distance for genotyped males and ungenotyped females**. Accuracy of QTL-EBV and total EBV for marker-assisted BLUP with neighboring marker (NM), 2-marker haplotype (HAP2) and 4-marker haplotype (HAP4), gene-assisted BLUP (GABLUP) when all animals are genotyped and conventional BLUP (CONBLUP); panels A and B: accuracy of QTL-EBV; panels C and D accuracy of total EBV; for MABLUP, sires and males in the last generation were genotyped, the rest was not genotyped, heritability is 0.30, the QTL explains 15% of the genetic variance, results are averages of 200 replicates.

Figure 3 shows the accuracy of QTL-EBV (panel A and B) and total EBV (Panel C and D) for genotyped males (panel A and C) and ungenotyped females (panel B and D) as a function of marker distance using three different haplotype methods for MABLUP or using CONBLUP or GABLUP when all animals were genotyped. For genotyped males (Figure 3A) the accuracy of the QTL-EBV was between 0.22 and 0.90 for NM, HAP2 and HAP4 and 1.0 for GABLUP. Among the three haplotype methods, HAP4 had the highest accuracy and NM the lowest. The accuracy decreased with increasing marker distance and more rapidly for HAP4 than for NM, due to a decreasing proportion of QTL variance explained by the haplotypes (Figure 1). For ungenotyped females (Figure 3B), the accuracy of the QTL-EBV was much lower than for genotyped males, between 0.15 and 0.57 for NM, HAP2 and HAP4, but with the same trends across marker distances as for genotyped animals. The MABLUP methods based on HAP2 and HAP4 were both able to increase substantially the accuracy of the total EBV of genotyped males in comparison to CONBLUP when the distance between the markers was small (Figure 3C). The accuracy of MABLUP with HAP4 approached the accuracy of gene-assisted BLUP when the marker distance was 0.1 cM or less. The advantage of MABLUP was negligible when the marker distance was large, e.g. 5 cM. For ungenotyped animals (Figure 3D), the increase in accuracy of total EBV of MABLUP over conventional BLUP was, however, negligible regardless of marker distance.

**Figure 4:**
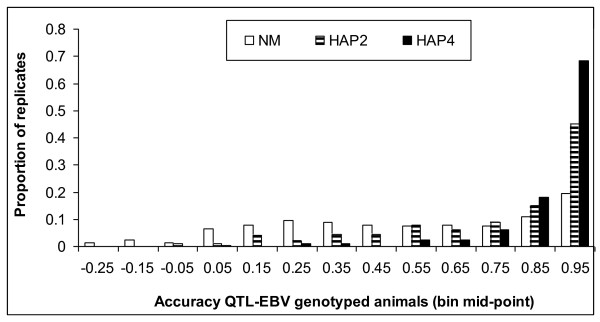
**Frequency distribution of accuracy of QTL-EBV of genotyped animals**. Proportion of replicates per 0.1-bin-class for accuracy of QTL-EBV of genotyped animals for neighboring marker (NM), 2-marker haplotype (HAP2) and 4-marker haplotype (HAP4); sires and males in last generation are genotyped, distance between markers is 0.1 cM, heritability is 0.3, the QTL explains 15% of the genetic variance, average of 200 replicates.

Although the average accuracy of QTL-EBV was moderate to high for genotyped males when markers were separated by 0.1 cM, substantial variation existed between replicates (Figure 4). Especially with NM, the variation between replicates was large and even negative accuracies were obtained, although in a very small proportion of the replicates (5.5% of replicates). With HAP4, accuracies of QTL-EBV were always positive and in 86.5% of the replicates larger than 0.80. With HAP2 this proportion equaled to 60% and with NM only to 30.5%. The figure clearly shows that HAP4 had not only the highest average accuracy, but also the least variation in accuracy of QTL-EBV.

#### Effect of heritability

Table 6 shows the accuracies of QTL-EBV, polygenic EBV and total EBV for genotyped males and ungenotyped females using different values of heritability in the three MABLUP methods when the marker distance was 0.1 cM. The accuracy of QTL-EBV increased with increasing heritability, as expected. However, the increase in accuracy of total EBV of MABLUP methods in comparison to CONBLUP was largest with a low heritability. For ungenotyped animals, the increase in accuracy with MABLUP in comparison to CONBLUP was smaller, e.g. from 0.35 to 0.37 with HAP4 at a heritability of 0.03, but the increase in accuracy was negligible when the heritability was 0.30. HAP4 had in all cases the highest accuracies for QTL-EBV, polygenic EBV and total EBV, i.e. the ranking of the methods did not change.

**Table 6:** Accuracies of QTL-EBV, polygenic EBV and total EBV for genotyped males and ungenotyped females

			Genotyped			Ungenotyped		
				
EBV	h^2^	CONBLUP	NM	HAP2	HAP4	NM	HAP2	HAP4
QTL	0.03		0.568	0.723	0.796	0.371	0.475	0.524
	0.10		0.542	0.770	0.865	0.349	0.493	0.554
	0.30		0.534	0.775	0.912	0.336	0.491	0.580
Polygenic	0.03		0.333	0.336	0.336	0.335	0.339	0.339
	0.10		0.444	0.452	0.456	0.454	0.444	0.452
	0.30		0.567	0.576	0.583	0.566	0.575	0.582
Total	0.03	0.351	0.387	0.407	0.418	0.362	0.368	0.371
	0.10	0.465	0.488	0.508	0.516	0.468	0.471	0.472
	0.30	0.594	0.605	0.616	0.622	0.595	0.596	0.596

Accuracies^1 ^of QTL-EBV, polygenic EBV and total EBV for different values of heritability for marker-assisted BLUP with neighboring marker (NM), 2-marker haplotype (HAP2) and 4-marker haplotypes (HAP4) and conventional BLUP (CONBLUP) (sires and males in last generation are genotyped; distance between markers is 0.1 cM, the QTL explains 15% of the genetic variance, results are averages of 200 replicates)

^1^Standard errors were between 0.007 and 0.022 for QTL-EBV, between 0.002 and 0.006 for polygenic EBV and between 0.002 and 0.005 for total EBV

Table 7 shows the regression of true on estimated breeding values for different values of heritability for the three MABLUP methods when the marker distance was 0.1 cM for genotyped males and ungenotyped females. The regressions for QTL-EBV were substantially lower than 1.0 in the majority of the situations, except when the heritability was 0.03. This indicated that the variance of the QTL-effect was overestimated when the heritability was 0.10 and 0.30. HAP4 had regression coefficients closest to 1.0 indicating that in this case, overestimation was the smallest. Regressions for polygenic and total EBV were in most cases close to one. The variances of the polygenic EBV were slightly overestimated in all cases. The variances of the total EBV were slightly overestimated for genotyped males for CONBLUP and MABLUP and slightly underestimated for ungenotyped females with MABLUP, but overestimated with CONBLUP. Overall, the variance of total EBV was less biased with MABLUP than with CONBLUP.

**Table 7:** Regression coefficients of estimated breeding values for genotyped males and ungenotyped females

			Genotyped			Ungenotyped		
				
EBV	h^2^	CONBLUP	NM	HAP2	HAP4	NM	HAP2	HAP4
QTL	0.03		0.867	1.115	1.143	0.797	1.109	1.165
	0.10		0.772	0.899	0.955	0.809	0.889	0.953
	0.30		0.869	0.909	0.917	0.744	0.884	0.910
Polygenic	0.03		0.945	0.962	0.970	0.948	0.965	0.975
	0.10		0.950	0.973	0.985	0.951	0.973	0.985
	0.30		0.951	0.966	0.976	0.954	0.965	0.973
Total	0.03	0.972	0.954	0.991	0.986	0.989	0.997	0.989
	0.10	0.987	0.981	0.975	0.974	1.022	1.014	1.011
	0.30	0.966	1.000	0.988	0.979	1.032	1.029	1.026

Regression^1 ^of true on estimated breeding values for QTL-EBV, polygenic EBV and total EBV for genotyped males and ungenotyped females for different values of heritability for marker-assisted BLUP with neighboring marker (NM), 2-marker haplotype (HAP2) and 4-marker haplotypes (HAP4) and conventional BLUP (CONBLUP) (sires and males in last generation are genotyped; distance between markers is 0.1 cM, the QTL explains 15% of the genetic variance, results are averages of 200 replicates)

^1^Standard errors were between 0.015 and 0.060 for QTL_EBV, between 0.004 and 0.015 for polygenic EBV and between 0.004 and 0.014 for total EBV; regressions where the variance of the predicted *nhc *was smaller than 0.0001 were omitted (denominator of regression coefficient)

Table 8 shows the bias in estimated breeding values for different values of heritability using the three MABLUP methods and CONBLUP for genotyped males and ungenotyped females when the marker distance was 0.1 cM. The polygenic EBV were on average biased upwards and the QTL-EBV were biased downwards, or in other words the QTL-effects were underestimated, but the polygenic EBV absorbed this effect. The total EBV were biased upwards for all methods when the heritability was 0.10 and 0.30, due to the shift of the estimated mean in the model, which was caused by genetic trend due to selection and the change in allele frequency of the QTL. Bias was largest for NM, whereas HAP2 and HAP4 were similar. Without selection total EBV were unbiased (results not shown). There was hardly any difference in bias between genotyped males and ungenotyped females. Adding the overall mean to the EBV removed the bias in total EBV. It can be concluded that total EBV of MABLUP and EBV of CONBLUP were biased due to selection, but this bias did no affect the ranking of animals.

**Table 8:** Bias in estimated breeding values for genotyped males and ungenotyped females

			Genotyped			Ungenotyped		
				
EBV	h^2^	CONBLUP	NM	HAP2	HAP4	NM	HAP2	HAP4
QTL	0.03		-0.008	-0.007	-0.001	-0.009	-0.007	-0.001
	0.10		-0.022	-0.013	0.000	-0.023	-0.014	-0.002
	0.30		-0.057	-0.028	0.008	-0.060	-0.032	0.003
Polygenic	0.03		0.006	0.002	-0.003	0.006	0.001	-0.003
	0.10		0.064	0.049	0.035	0.064	0.049	0.035
	0.30		0.125	0.086	0.053	0.126	0.087	0.055
Total	0.03	0.007	-0.002	-0.005	-0.004	-0.003	-0.006	-0.005
	0.10	0.042	0.041	0.035	0.034	0.041	0.035	0.034
	0.30	0.036	0.068	0.058	0.061	0.067	0.055	0.058

Bias^1 ^(estimated - true breeding value) in QTL-EBV, polygenic EBV and total EBV for genotyped males and ungenotyped females for different values of heritability for marker-assisted BLUP with neighboring marker (NM), 2-marker haplotype (HAP2) and 4-marker haplotypes (HAP4) and conventional BLUP (CONBLUP) (sires and males in last generation are genotyped; distance between markers is 0.1 cM, the QTL explains 15% of the genetic variance, results are averages of 200 replicates)

^1^Standard errors were between 0.003 and 0.012 for h^2 ^= 0.03, between 0.005 and 0.025 for h^2 ^= 0.10 and between 0.009 and 0.037 for h^2 ^= 0.30

## Discussion

In this study we developed a method to predict haplotypes of ungenotyped animals using pedigree information of genotyped animals in mixed model equations and we evaluated the use of these predicted haplotypes in marker-assisted BLUP. The method is an extension of Gengler *et al*. [14,15] to multi-allelic markers or haplotypes. The method was evaluated with Monte Carlo simulation. Clearly the predicted number of haplotype copies was regressed towards the mean and more so than the gene contents in Gengler *et al*. [14,15], especially when the frequency of a certain haplotype was low, which is more likely with longer haplotypes because of an increasing number of haplotypes. When using only a neighbor marker, the predicted gene contents were in the same range as in Gengler *et al*. [14,15]. Because of the almost-unity heritability the number of haplotype copies is hardly regressed towards the mean for genotyped animals. The accuracy of the predicted haplotypes was lower for HAP4 than for HAP2 and decreased with increasing marker distance due to the increased probability of recombination. Lowering the heritability might be an option, taking into account that the number of haplotype copies from parent to offspring is not fully heritable but subject to recombination. However, BLUP is very robust against changes in heritability and preliminary results showed no effect when the heritability was changed to 0.95.

The 4-marker haplotype gave the best results in marker-assisted breeding value estimation. It captured 90% of the QTL-variance when markers were separated by 0.1 cM. Because of this high proportion of explained QTL-variance, the proportion of QTL-variance explained by the haplotype can not increase much, and therefore we did not consider longer haplotypes. Furthermore, longer haplotypes are more subject to recombination, decreasing the accuracy of predicted number of haplotype copies. Hayes *et al*. [6] found that 6-marker haplotypes explained more QTL-variance than 4-marker haplotypes, but had much lower proportions of QTL-variance explained by the markers due to lower marker density and lower LD. Hayes *et al*. [6] found that the increase in accuracy was much higher with haplotypes than with using a neighbor marker in agreement with this study. Calus *et al*. [22] investigated the use of different definitions of haplotypes on the accuracy of genomic selection and found that with a high marker density the regression on single SNP worked almost as well as haplotypes with two markers. In their study all SNP were used for a single SNP regression, whereas in this study only one SNP was used to estimate the QTL-effect. This disfavored the neighbor marker method in our study, although the ranking of the alternatives is the same as in Calus *et al*. [22]. In the context of QTL fine-mapping, Grapes *et al*. [23] found that single marker regression with 10 markers performed worse than an IBD-method using linkage disequilibrium and linkage analysis information with a haplotype window of 10 markers, but single marker regression performed similarly when 20 markers were used. Zhao *et al*. [24] found that the power of a model with regression on two or four SNP yielded higher power to detect QTL than 2- or 4-marker haplotypes. This suggests that ranking of methods for QTL mapping might be different than for accuracy of marker-assisted or genomic selection [25].

The proportion QTL-variance explained by the haplotypes or the neighbor marker (*r*^2^) was higher than in Hayes *et al*. [6]. At marker distances ranging from 0.1 to 1.0 cM, estimated *r*^2 ^in cattle populations have been found lower (~0.05 - 0.27) than those found in this simulation study [6,26-28]. However, in pig and poultry populations higher *r*^2 ^have been estimated (~0.20-0.50 in pigs and poultry) [29,30], resembling the observed *r*^2 ^in our study. The *r*^2 ^between neighbor marker and QTL or between pairs of markers followed the expected *r*^2 ^based on distance in cM and the effective population size [31]. The lower *r*^2 ^values found at short distance in cattle populations is probably due to much higher effective population sizes in the past, because LD at short distances reflects more the past effective population size [32]. As a consequence of lower LD at short distances in cattle, a higher SNP density than that used in this study is necessary to achieve in cattle the same accuracy of QTL-EBV as presented here.

Haplotypes were assumed to be unrelated in this study and it was assumed that the same QTL-allele is linked to a certain haplotype (identity-by-state = IBS). Due to recombination, linkage phases between haplotypes and QTL may be different in different families. In the context of genomic selection, Calus *et al*. [22] compared 2-marker IBS-haplotypes with 2- and 10-marker identity-by-descent haplotypes using combined linkage disequilibrium linkage analysis information (LDLA) to construct the inverse IBD-matrices. They found that IBD-haplotypes yielded higher accuracies, especially when using 10-marker windows, but at the cost of much higher computing time. The difference between IBS and IBD-haplotypes decreased with increasing marker density. Therefore, in our study it is unlikely that IBD-haplotypes would increase accuracy significantly when the distance between the markers is less than 0.1 cM.

A major disadvantage of using haplotypes is the need to phase the data. Hayes *et al*. [6] estimated the effect of haplotyping errors on the proportion of QTL-variance explained by the haplotypes in their data set and found a limited effect, but suggested that phasing errors are dependent on the data structure used. Accurate and fast algorithms are available for use in livestock populations [33,34,28]. Windig and Meuwissen [34] have shown that their algorithm is very fast and yields almost perfect haplotype reconstruction with dense marker maps in pedigreed populations. Its performance was similar to that of SIMWALK2 [35] in terms of accuracy, but with a much lower computing time. Furthermore, the presented method can accommodate haplotyping errors, e.g. by adjusting the heritability of *nhc *to a lower value, albeit at the expense of a lower accuracy.

The major advantage of the method used in this study is its computing efficiency, because optimized BLUP software can be used to predict haplotypes. The computation time was respectively ~4, 6 and 10 s for neighboring marker (NM), 2-marker haplotypes (HAP2) and 4-marker haplotypes (HAP4) to predict the genotypes/haplotypes on a dual-processor 64-bit Windows PC with 2.40 GHz and 36 GB of RAM; programs were compiled for 32-bit. Therefore, breeding companies do not need other software for imputing genotypes, which is usually much slower and much more memory intensive, prohibiting its use for large populations, e.g. with more than a million animals. An additional advantage is that no assumptions are needed on where ungenotyped animals should appear in the pedigree, it can handle all possible scenarios. Therefore, the proposed method is very suitable for application of marker-assisted breeding value estimation in large populations, such as national evaluations in cattle. Also for genomic selection purposes the method is very useful, e.g. for 50,000 SNP-markers it would take only about two days on a single processor to predict all SNP-genotypes or haplotypes for a similar number of animals as in this study.

The use of 4-marker haplotypes (HAP4) increased the accuracy of marker-assisted breeding value estimation substantially in comparison to conventional breeding value estimation for genotyped animals, but the benefit for ungenotyped animals was small in agreement with Mulder *et al*. [8]. However, with a low heritability, ungenotyped animals gained considerably in accuracy. This can be visualized by approximating the accuracy of the total EBV (*r*_*totalEBV*_) as:(5)

where *q*^2 ^is the proportion of genetic variance explained by the haplotypes (= , where  is the accuracy of the QTL-EBV and *Q*^2 ^is the amount of genetic variance explained by the QTL),  is the accuracy of the polygenic EBV and *r*_*h *_is the accuracy of the predicted number of haplotype copies. If we take the situation where the heritability is 0.03, the distance between markers is 0.1 cM and the QTL explains 15% of the genetic variance,  is 0.34 (Table 6) and we assume that *q*^2 ^is 0.10 (assuming  = 0.8 (Table 6)), then Equation (5) yields *r*_*totalEBV *_= 0.374, close to the value in Table 6. Using Equation (5), we can also quantify the benefit of genome-wide EBV for ungenotyped animals. Lets assume that we can explain 90% of the genetic variance by markers (*q*^2 ^= 0.9), then we can increase *r*_*totalEBV *_up to 0.58 assuming that  is constant. So even for ungenotyped animals genome-wide EBV can increase accuracy in comparison to conventional BLUP, especially for low heritability traits, when their paternal ancestors are genotyped.

## Conclusions

In this study we show that mixed model equations can be used to predict number of haplotype copies for ungenotyped animals and these predicted number of haplotype copies can be used in marker-assisted breeding value estimation. Four-marker haplotypes give the highest accuracy for total estimated breeding values. The accuracy of the total EBV increases for genotyped animals, but for ungenotyped animals the increase is marginal unless the heritability is smaller than 0.1. The method works best when the distance between the markers is less than 1 cM. The proposed method is computationally very efficient and suitable to apply for marker-assisted breeding value estimation in large livestock populations including effects of a number of known QTL. Marker-assisted breeding value estimation using predicted haplotypes increases accuracy especially for traits with low heritability. It is expected that genomic selection for ungenotyped animals using predicted haplotypes or marker genotypes will be beneficial especially for low heritable traits.

## Competing interests

The authors declare that they have no competing interests.

## Authors' contributions

HAM developed the method, ran the simulations and evaluations and drafted the manuscript. MPLC and RFV discussed the method and results and helped to draft the manuscript. All authors read and approved the final manuscript.

## Appendix

### Derivation of haplotype variance used in mixed models

Assuming that the haplotypes explain 100% of the QTL-variance, the variance of haplotype effects  used in Equation (4) can be calculated similarly to the variance when regressing on one bi-allelic marker/QTL:(A1)

where *α *is the allele substitution effect, *p *is the allele frequency of one of the two SNP-alleles. Extrapolating the result of Equation (A1) to *n *haplotypes yields:(A2)

where *m*_*i *_is the frequency of haplotype *i*. Assuming equal frequencies of all *n *haplotypes yields:(A3)

The limit of Equation (A3) is:(A4)

showing that the variance of haplotype *i *is half the additive genetic variance of the QTL with an infinite number of haplotypes. Although the result in Equation (A2) depends on haplotype frequencies and number of haplotypes, preliminary analyses showed that using the result of Equation (A4) yields high accuracies of QTL-EBV. Furthermore, these preliminary analyses showed that the accuracy of the QTL-EBV is insensitive to .
